# KAT7 promotes radioresistance through upregulating PI3K/AKT signaling in breast cancer

**DOI:** 10.1093/jrr/rrac107

**Published:** 2023-02-01

**Authors:** Yan Ma, Xiaohua Chen, Ting Ding, Hanqun Zhang, Qiuning Zhang, Huanyu Dai, Haibo Zhang, Jianming Tang, Xiaohu Wang

**Affiliations:** The First School of Clinical Medicine, Lanzhou University, Lanzhou, Gansu, 730000, P. R. China; Department of Radiation Oncology, The First Hospital of Lanzhou University, Lanzhou University, Lanzhou, Gansu, 730000, P. R. China; Department of Radiation Oncology, The First Hospital of Lanzhou University, Lanzhou University, Lanzhou, Gansu, 730000, P. R. China; Department of Endocrinology, Yiyang Central Hospital, Yiyang, Hunan, 413000, P. R. China; The First School of Clinical Medicine, Lanzhou University, Lanzhou, Gansu, 730000, P. R. China; Department of Oncology, Guizhou Provincial People's Hospital, Guiyang, Guizhou, 550002, P.R. China; Institute of Modern Physics, Chinese Academy of Sciences, Lanzhou, Gansu, 730000, P.R. China; Lanzhou Heavy Ion Hospital, Lanzhou, Gansu, 730000, P.R. China; Department of Oncology, The First Hospital of Lanzhou University, Lanzhou University, Lanzhou, Gansu, 730000, P. R. China; Oncology Center, Department of Radiation Oncology, Zhejiang Provincial People's Hospital, Affiliated People's Hospital, Hangzhou Medical College, Hangzhou, Zhejiang, 310014, P. R. China; Department of Radiation Oncology, The First Hospital of Lanzhou University, Lanzhou University, Lanzhou, Gansu, 730000, P. R. China; Key Laboratory of Biotherapy and Regenerative Medicine of Gansu Province, The First Hospital of Lanzhou University, Lanzhou University, Lanzhou, Gansu, 730000, P. R. China; The First School of Clinical Medicine, Lanzhou University, Lanzhou, Gansu, 730000, P. R. China; Institute of Modern Physics, Chinese Academy of Sciences, Lanzhou, Gansu, 730000, P.R. China; Lanzhou Heavy Ion Hospital, Lanzhou, Gansu, 730000, P.R. China

**Keywords:** Lysine acetyltransferase 7 (KAT7), PI3K/AKT signaling, radioresistance, breast cancer

## Abstract

Chromatin-modifying enzymes are commonly altered in cancers, but the molecular mechanism by which they regulate cancers remains poorly understood. Herein, we demonstrated that Lysine acetyltransferase 7 (KAT7) was upregulated in breast cancer. KAT7 expression negatively correlated with the survival of breast cancer patients, and KAT7 silencing suppressed breast cancer radioresistance *in vitro*. Mechanistically, KAT7 activated Phosphatidylinositol-4,5-bisphosphate 3-kinase catalytic subunit alpha (PIK3CA) transcription, leading to enhanced PI3K/AKT signaling and radioresistance. Overexpression of AKT or PIK3CA restored radioresistance suppression induced by KAT7 inhibition. Moreover, overexpression of KAT7, but not KAT7 acetyltransferase activity-deficient mutants promoted AKT phosphorylation at the Ser473 site, PIK3CA expression and radioresistance suppression due to KAT7 inhibition. In conclusion, KAT7 has huge prospects for clinical application as a new target for predicting radioresistance in breast cancer patients.

## INTRODUCTION

Breast cancer is the most common malignant tumor in women, with a 5-year survival rate of more than 80% [1, 2]. Overwhelming evidence substantiates that the epigenetic landscape is abnormally expressed in cancer partially due to aberrant histone modifications and mutations [3–5]. However, the molecular mechanism of how histone modifiers regulate radioresistance remains poorly elucidated.

Lysine acetyltransferase 7 (KAT7, also named MYST2/HBO1) is a member of the MYST KAT family, which can control cell survival, DNA replication and transcription [6–8]. KAT7 was reported to interact with the origin of replication [9]. KAT7 was also involved in several replication-associated processes via interacting with replication factors [10, 11]. Deleted KAT7 in cell lines was proven to stall DNA replication [12]. KAT7 inactivation led to a global loss of H3K14ac and decreased expression of a broad range of genes, including patterning genes required for the normal development of postgastrulation embryos, which is correlated with decreased cell survival but no alteration in cell proliferation or DNA replication *in vitro* or *in vivo* [13]. Over the years, KAT7 aberrant expression has been associated with oncogenesis in gastric cancer [14], acute myeloid leukemias [15], bladder cancer [16] and ovarian cancer [17]. Ample evidence suggests that KAT7 can form various MYST complexes subunits, including human MYST/Esa1 associated factor 6 (hEAF6), Bromodomain and PHD finger containing 1/2/3 (Brpf1/2/3), Inhibitor of growth family member 4/5 (ING4/5) and Jade family PHD finger 1/2/3 (JADE1/2/3), to regulate its acetyltransferase activity [8, 18–21]. Moreover, KAT7-containing MYST complexes have been localized in the promoter and intragenic region of target genes and play an essential functional role at these sites [8, 22]. In addition, KAT7 acetylates histone H3K14 and H3K23 via binding BRPF, whereas KAT7 acetylates histone H4K5, H4K8 and H4K12 via binding JADE [7, 18–21]. Mounting evidence suggests that KAT7 suppression leads to H3K14ac reduction in mouse embryos and fetal liver erythroblasts [7, 14]. Nonetheless, the mechanisms by which KAT7 regulates oncogenesis and radioresistance in breast cancer remain unclear, warranting further investigation.

Herein, we reported a hitherto undocumented relationship between KAT7 expression in breast cancer and its relationship with survival. Then we examined the function of KAT7 in radioresistance *in vitro*. Finally, we assessed the molecular mechanisms by which KAT7 mediates breast cancer radioresistance.

## MATERIALS AND METHODS

### Cell lines

Breast cancer cells MCF7 (wild type P53), MDA-MB-231 (mutant type P53) and BT549 (mutant type p53) were obtained from the Cell Bank of the Chinese Scientific Academy (Shanghai, China). Cells were cultured in RPMI-1640 (Invitrogen, Carlsbad, CA) supplemented with 10% fetal bovine serum (Gibco, Life Technologies, CA) in a humidified incubator with 5% CO_2_ at 37°C. Normal mammary epithelial cell line MCF10A was cultured in DMEM/F12 (Invitrogen) supplemented with 20 ng/ml epidermal growth factor, 5% horse serum, 0.5 μg/ml hydrocortisone, 10 μg/ml insulin, 100 ng/ml cholera toxin and 100 μg/ml penicillin-streptomycin.

### Plasmids

Phosphatidylinositol-4,5-bisphosphate 3-kinase catalytic subunit alpha (PIK3CA), KAT7 and Myr-AKT vectors were purchased from Addegen: PI3KC2alpha- PX (Plasmid #119119, pGEX4T2 vector), pFETCh_KAT7 (Plasmid #86266, pFETCh_ Donor vector), pENTR-myr-AKT-HA (Plasmid #31790, pENTR vector). The shRNAs were designed as follows: shKAT7–1 (5’-GGAGAAGTTAAGGCTGCAAGG-3’); shKAT7–2 (5’-GCTATGGCAAGATGCTTATTG-3’). KAT7E508Q point mutation were performed using a site-directed mutagenesis kit (Invitrogen) according to the manufacturer’s protocol. shC is control group with empty vectors. The production of shKAT7 cells is stable transfection. Cancer cells were infected using lentivirus expressing shRNAs with 8 μg/ml polybrene. Infected cells were selected by treatment with 5 μg/ml puromycin 48–72 h after infection. Multiple monoclonal cultures were screened for shRNAs by Western blotting (WB) and RT-PCR analysis.

### RNA extraction and qRT-PCR

Trizol reagent (Takara, Dalian, China) was used to extract the total RNA from cells. The Reverse Transcription Kit (Takara, Dalian, China) was used to synthesize cDNAs. The qPCR Master Mix (SYBR Green) (Clontech, USA) was used for qPCR reactions. GAPDH was used as a control. The primers were designed as follows: 5’-ATTCTGGACTGAGCAAAGA ACAG-3’ (KAT7-forward); 5’-GTCATACTCGCTTGT CAGGTTTT-3’ (KAT7-reverse); 5’-CCACGACCATCATCAGGTGAA-3’ (PIK3CA- forward); 5’-CCTCACGGAGGCATTCTAAAGT-3’ (PIK3CA-reverse); 5’-GGAGCGAG ATCCCTCCAAAAT-3’ (GAPDH-forward); 5’-GGCTGTTGTCATACTTCTCATGG-3’ (GAPDH-reverse).

### Western blot analysis

WB experiments were performed as previously described [21]. Antibodies were purchase as follows: GAPDH (ab8245; 1:5000; Mouse monoclonal; Abcam); KAT7 (ab37289; 1:1000; Rabbit polyclonal; Abcam); phospho-AKT (#4060S; 1:1000; Rabbit polyclonal; Cell Signaling Technology); AKT (#9272S; 1:1000; Rabbit monoclonal; Cell Signaling Technology); PIK3CA (4F3; 1:1000; Mouse monoclonal; ThermoFisher Scientific).

### Luciferase promoter assay

We used pGL3-PIK3CA promoter plasmids and Lipofectamine 3000 transfection reagent (Invitrogen) for transfection. The control group used the pRL Renilla luciferase vector (Promega). To detect the luciferase signals, we used a dual-luciferase Reporter kit (Promega).

### Cell proliferation and colony formation

For cell proliferation detection, the cancer cells (1000 cells/well) were seeded in 96-well plates and then detected using a WST-1 Assay Kit (Roche) for succession days. For colony formation, the cancer cells (500 cells/well) were seeded into the 6-well plates for 2 weeks, and then 1% crystal violet solution was used to stain all cell colonies. Finally, we take pictures for colonies and count the numbers of colonies.

### Irradiation

A 6 MV X-ray linear accelerator with a 200 cGy/min dose rate (Primus, Siemens AG, Erlangen, Germany) was used for radiation. The dose of radiation is 4Gy.

### Statistical analysis

Statistical analyses were performed using the GraphPad Prism version 5.0 software (GraphPad). One-way analysis of variance was used to analyze clonogenic survival. The significance analysis of data between two groups was assessed by a two-tailed paired Student’s t-test. A *P*-value < 0.05 was statistically significant.

## RESULTS

### KAT7 expression has prognostic value for breast cancer

**Fig. 1:**
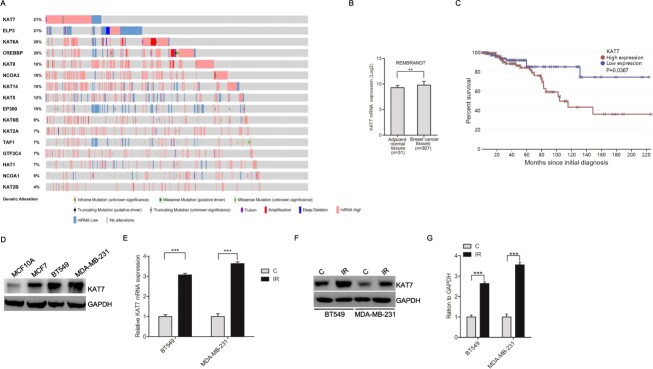
KAT7 expression is prognostic for breast cancer. **A,** Representative mRNA high or low proportion of KAT family. Data were downloaded from TCGA. Light salmon represents mRNA high proportion, whereas dark blue represents mRNA low proportion. **B**, Representative KAT7 expression in breast cancer tissues and adjacent normal tissues. Data were downloaded from the REMBRANDT data set and presented as mean ± SEM. A two-tailed t-test was used to analyze the significance, ^*^^*^P < 0.01. **C**, KAT7 expression levels were negatively associated with overall survival. Data were downloaded from the REMBRANDT data set. **D**, Representative KAT7 expression in breast cancer cells and MCF10A. **E** and **F**, KAT7 mRNA and protein are upregulated in ionizing radiation cells. **G**, Quantification of KAT7 protein in **F**. EV represents empty vector. Error bars in **E** and **G** represent the mean ± S.D. ^*^^*^^*^P < 0.001. Data are representative of three independent experiments.

To uncover if KAT7 is essential for breast cancer progression, we first analyzed alterations in the mRNA levels of the KAT family in breast cancer patients. We downloaded the cancer genome atlas (TCGA) data set and found that proportion of KAT7 mRNA high exhibited the highest in the KAT family (Fig. 1A). Using the REMBRANDT data set (http://www.betastasis.com), we found that KAT7 had a higher mRNA expression in breast tumor tissues than in the adjacent normal tissues (Fig. 1B). It was the same tendency indicated between normal (MCF10A) and cancer cells (MCF7, BT549 and MDA-MB-231) (Fig. 1D). As shown in Fig. 1C, KAT7 expression levels were negatively associated with overall survival by Kaplan–Meier plotter database analysis. Finally, at the time of 48 hours after receiving irradiation, we checked KAT7 expression and demonstrated that irradiation could induce KAT7 mRNA and protein expression (Figs 1E–1G).

### KAT7 increases radioresistance in breast cancer cells

**Fig. 2:**
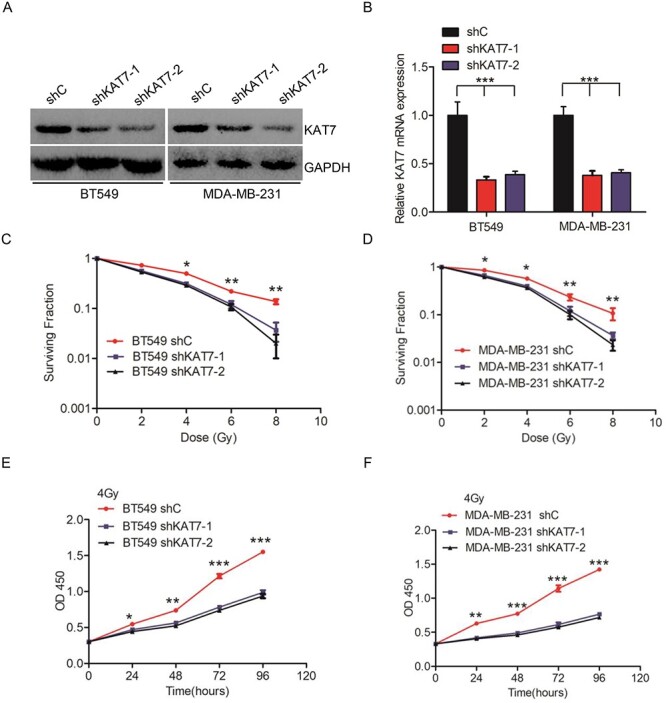
KAT7 increases radioresistance in breast cancer cells. **A–B**, Representative KAT7 protein and mRNA expression in KAT7 knockdown breast cancer cells. **C–D**, KAT7 inhibition significantly suppresses cell survival fraction at doses of 2, 4, 6 and 8 Gy with cells were cultured for 2 weeks. **E–F**, KAT7 knockdown suppressed cell proliferation at 0, 24, 48, 72 and 96 hours after 4 Gy irradiation. EV represents empty vector. Error bars in **B**, **C**, **D** and **E** represent the mean ± S.D. ^*^P < 0.05. ^*^^*^^*^P < 0.001. ^*^^*^^*^P < 0.001. Data are representative of three independent experiments.

To detect the role of KAT7 in breast cancer radioresistance, we first suppressed KAT7 protein and mRNA expression using shRNAs (Figs 2A–2B). We found that KAT7 inhibition significantly suppressed the cell survival fraction at doses of 2, 4, 6 and 8 after 2 weeks (Figs 2C–2D). Furthermore, KAT7 knockdown suppressed cell proliferation at 0, 24, 48, 72 and 96 hours after 4 Gy irradiation (Figs 2E–2F). Taken together, these results provided compelling evidence that KAT7 promoted radioresistance in breast cancer.

### KAT7 regulates AKT activity

**Fig. 3:**
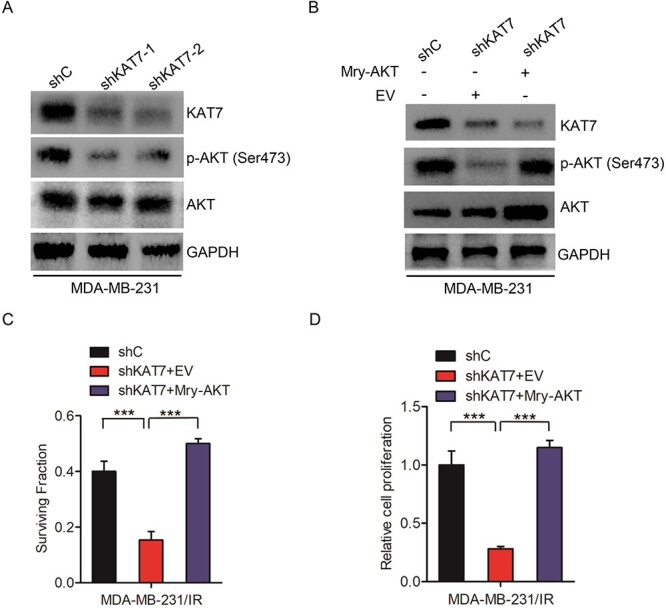
KAT7 regulates AKT activity. **A**, Effects of KAT7 knockdown on AKT phosphorylation at the Ser473 site in breast cancer cells. **B**, Effects of Myr-AKT overexpression on KAT7 knockdown-inhibited AKT activation. **C–D**, Myr-AKT overexpression rescued KAT7 knockdown-inhibited surviving fractions and cell proliferation after irradiation. EV represents empty vector. Error bars in **C** and **D** represent the mean ± S.D. ^*^^*^^*^P < 0.001. Data are representative of three independent experiments.

Given that the AKT signaling pathway is critical for radioresistance in breast cancer [24, 25], we first analyzed the effect of KAT7 suppression on AKT phosphorylation in breast cancer cells. As shown in Fig. 3A and Supplementary Fig. 1, KAT7 knockdown significantly inhibited AKT phosphorylation at Ser473 site, which indicated that KAT7 regulates AKT activity in breast cancer. To further assess whether KAT7 regulates AKT activity, activated AKT mutant (Myr-AKT) was overexpressed in KAT7 knockdown cells. As shown in Fig. 3B, overexpression of Myr-AKT rescued AKT phosphorylation at the Ser473 site inhibited by KAT7 knockdown. Moreover, Myr-AKT overexpression restored KAT7 suppression-inhibited cell survival fraction and proliferation after irradiation (Figs 3C–3D). These results indicated that KAT7 promoted radioresistance by mediating AKT activity.

### KAT7 activates PI3K/AKT signaling by upregulating PIK3CA expression in breast cancer

**Fig. 4:**
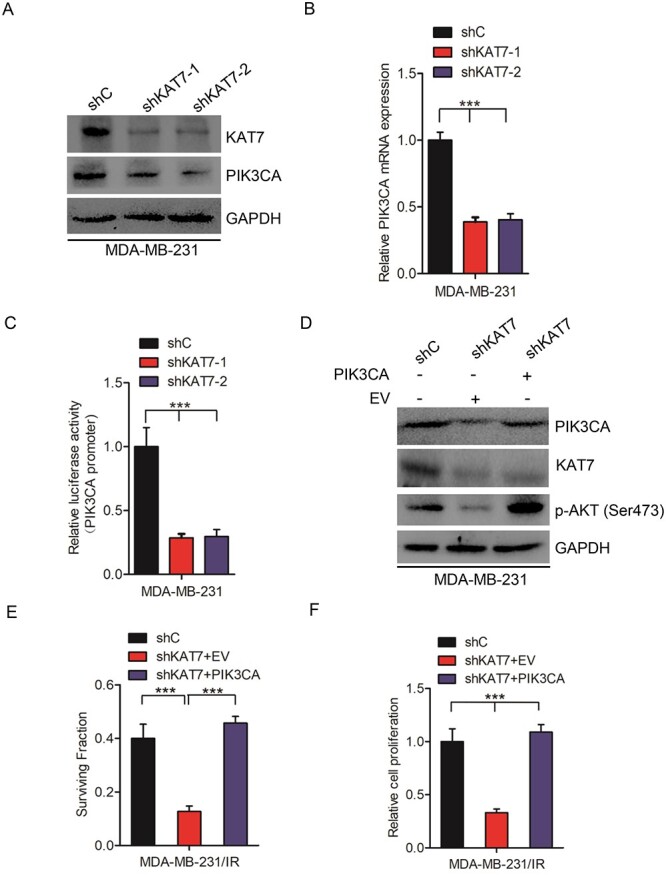
KAT7 activates PI3K/AKT signaling by upregulating PIK3CA expression in breast cancer. **A–B**, Effects of KAT7 knockdown on PIK3CA protein and mRNA expression in breast cancer cells. **C**, Luciferase assay of KAT7 knockdown inhibiting PIK3CA promoter activity in MDA-MB-231 cells. **D**, Effects of PIK3CA overexpression on KAT7 knockdown-inhibited AKT activation. **E–F**, PIK3CA overexpression rescued KAT7 knockdown-inhibited surviving fractions and cell proliferation after irradiation. EV represents empty vector. Error bars in **B**, **C**, **E** and **F** represent the mean ± S.D. ^*^^*^^*^P < 0.001. Data are representative of three independent experiments.

The phosphatidylinositol-4-5-bisphosphate-3-kinase catalytic subunit-α (PIK3CA) gene encodes the p110α subunit of class IA PI3K to phosphorylate phosphatidylinositol-4,5-bisphosphate (PIP2) and converts it to phosphoinositide 3,4,5 trisphosphate (PIP3), and then induces sustained activation of AKT through the PI3K/AKT/mTOR pathway [26]. To investigate the role of PIK3CA in KAT7 regulated PI3K/AKT signaling, we first detected PIK3CA expression in KAT7 knockdown cells. As shown in Figs 4A and 4B, PIK3CA protein and mRNA expression were downregulated in KAT7 knockdown groups compared with the control group. Promoter luciferase assays showed that KAT7 knockdown suppressed PIK3CA activity (Fig. 4C). As shown in Supplementary Fig. 1, KAT7 knockdown also suppressed PIK3CA protein in BT549. Overall, these findings demonstrated that KAT7 regulates PIK3CA expression in breast cancer.

To further assess whether KAT7 activates PI3K/AKT signaling by upregulating PIK3CA expression in breast cancer, PIK3CA was overexpressed in KAT7 knockdown cells. As shown in Figs 4D, 4F and Supplementary Fig. PIK3CA overexpression restored KAT7 suppression-inhibited AKT phosphorylation at the Ser473 site, radiation cell survival fraction and radiation cell proliferation at the dose of 4 Gy in MDA-MB-231 and BT549 cells. In conclusion, KAT7 activates PI3K/AKT signaling to regulate radioresistance by upregulating PIK3CA expression in breast cancer.

### KAT7 acetyltransferase activity is required for radioresistance in breast cancer

**Fig. 5:**
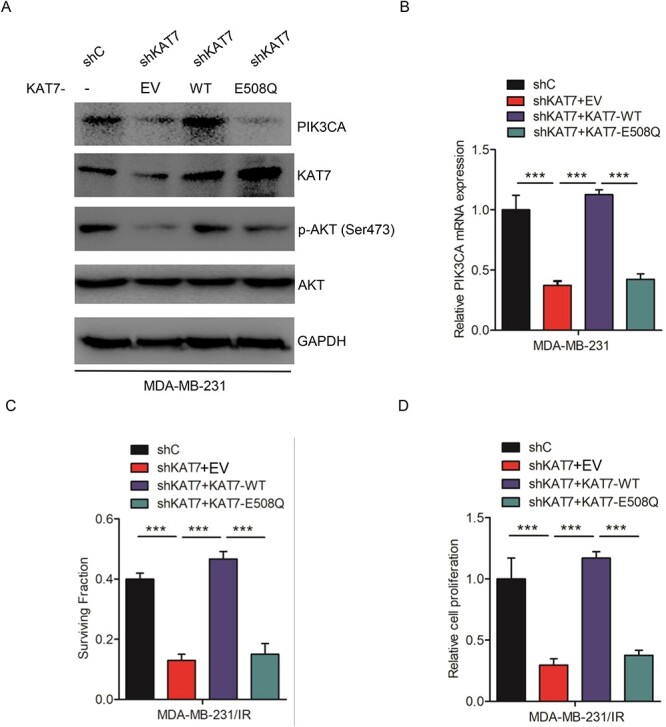
KAT7 acetyltransferase activity is required for radioresistance in breast cancer. **A**, Effects of KAT7^WT^ and KAT7^E508Q^ overexpression on KAT7 knockdown-inhibited AKT activation and PIK3CA protein. **B**, KAT7^WT^ overexpression restored KAT7 knockdown-inhibited PIK3CA mRNA, whereas KAT7^E508Q^ overexpression did not alter them. **C–D**, Effects of KAT7^WT^ and KAT7^E508Q^ overexpression on shKAT7–1-inhibited surviving fractions and cell proliferation after irradiation. EV represents empty vector. Error bars in **B**, **C** and **D** represent the mean ± S.D. ^*^^*^^*^P < 0.001. Data are representative of three independent experiments.

To explore whether KAT7 acetyltransferase activity is necessary for PI3K/AKT signaling activation, we first constructed KAT7 wild type vector (KAT7^WT^) and acetyltransferase activity deficient mutant type vector (KAT7^E508Q^). As shown in Figs 5A–5B, KAT7^WT^ overexpression restored shKAT7–1-inhibited AKT phosphorylation at the Ser473 site and PIK3CA expression, whereas KAT7^E508Q^ overexpression did not induce alterations. Moreover, cell survival fraction and proliferation at the dose of 4 Gy. exhibited similar effects (Figs 5C–5D). Taken together, KAT7 acetyltransferase activity is required for activating the PI3K/AKT signaling pathway to regulate radioresistance in breast cancer.

## DISCUSSION

Except breast cancer [27], KAT7 was reported to function as an oncogene in other cancers, including gastric cancer [14], acute myeloid leukemias [15], bladder cancer [16] and ovarian cancer [17]. However, the function and mechanism of KAT7 in breast cancer radioresistance remain largely understudied. Herein, we revealed that KAT7 upregulates PIK3CA, leading to activation of the PI3K/AKT signaling pathway, thus promoting radioresistance in breast cancer.

Our results revealed that KAT7 acts as an oncogene in breast cancer radioresistance. Consistently, a previous study reported that KAT7 promotes destabilization of estrogen receptor α through lysine 48-linked ubiquitination to induce breast cancer cell proliferation [28]. KAT7 has recently been reported to function as a novel cyclin E/CDK2 substrate that enriches stem-like cells in breast cancer [29]. The level of KAT7 is associated with clinical outcomes in cancer patients, suggesting its value with clinical prognosis, histological grading and therapeutic targets in breast cancer [30, 31]. KAT7 enhances growth of breast cancer cells and knockdown of the KAT7 gene impairs cell proliferation, which suggests that KAT7 function as an oncogene [32]. Phosphorylation of downstream KAT7 by the LMW-E/CDK2 complex enhances the self-renewal ability of breast cancer cells and the enrichment of cancer stem cells populations [33]. Also, KAT7 contributes to the ubiquitination of ERα *in vivo* thereby destabilizing it [34]. KAT7 is a potential prognostic marker and therapy target for breast cancer. Besides, intensity-modulated radiation therapy is widely used for breast cancer [35]. Current evidence suggests that ionizing radiation alters characteristics such as gene epigenetics and gene expression, which lead to radioresistance [36]. Moreover, radioresistance has also been reported to hamper the therapeutic efficacy of breast cancer [37]. The present study found that KAT7 expression is upregulated in breast cancer compared with normal tissue. Furthermore, we found a negative correlation between KAT7 expression and survival of breast cancer patients. Suppression of KAT7 by shRNAs inhibited breast cancer radioresistance. These data demonstrate that KAT7 is crucial for breast cancer tumorigenesis.

Moreover, our data revealed that KAT7 regulates radioresistance through the PI3K/AKT signaling pathway. It has been reported that KAT7 mediates CENP-A chromatin by antagonizing Suv39h1-mediated centromere inactivation [38]. Interestingly, KAT7 promotes cell proliferation and development by associating with the scaffolding protein JADE1 [39]. Our results demonstrate that KAT7 knockdown inhibited breast cancer radioresistance through PI3K/AKT signaling and PIK3CA or Mry-AKT overexpression rescued KAT7 inhibition-suppressed cell radioresistance. Moreover, KAT7 could upregulate PIK3CA transcription, leading to sustained activation of the PI3K/AKT signaling pathway. In conclusion, our data reveal that KAT7 mediates cell radioresistance by activating the PI3K/AKT signaling pathway.

In a nutshell, our results corroborated that KAT7 has significant value as a novel molecular target for treating breast cancer. The present study found that KAT7 mediates radioresistance via activating the PI3K/AKT signaling pathway. The new roles of KAT7 in breast cancer radioresistance contribute to a better understanding of KAT7 functions and regulation in cancer.

## Supplementary Material

Supplementary_Figure_rrac107

## Data Availability

All data generated or analyzed during this study are included in this manuscript.
