# Correction to: KAT7 promotes radioresistance through upregulating PI3K/AKT signaling in breast cancer

**DOI:** 10.1093/jrr/rrad019

**Published:** 2023-03-20

This is a correction to: Yan Ma, Xiaohua Chen, Ting Ding, Hanqun Zhang, Qiuning Zhang, Huanyu Dai, Haibo Zhang, Jianming Tang, Xiaohu Wang, KAT7 promotes radioresistance through upregulating PI3K/AKT signaling in breast cancer, *Journal of Radiation Research*, 2023;, rrac107, https://doi.org/10.1093/jrr/rrac107

In the originally published version of this manuscript, Yan Ma's name was incorrectly written as “Ya Ma”.

Panels B to G from Figure 1 were missing.

These errors have now been corrected.

