# Supplementary material for: KAT7 promotes radioresistance through upregulating PI3K/AKT signaling in breast cancer
Source: J Radiat Res. 2023 Feb 1;64(2):448–56. doi: 10.1093/jrr/rrac107 (PMC10036104; doi:10.1093/jrr/rrac107)
Supplement: Supplementary_Figure_rrac107 [file supplementary_figure_rrac107.pptx]

## Slide 1
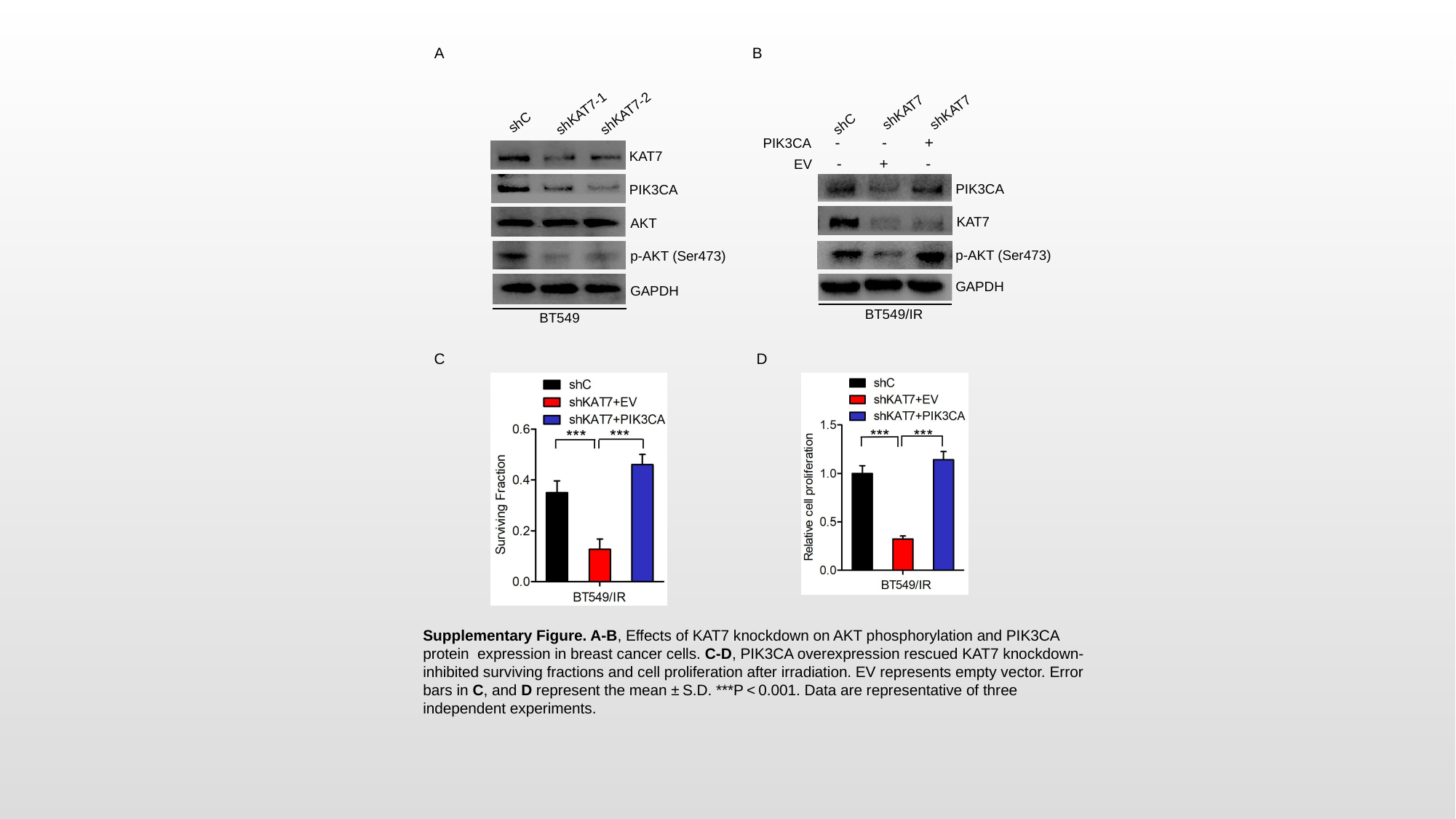

A
 B
shKAT7
shKAT7
shKAT7-1
shKAT7-2
shC
shC
PIK3CA - - +
KAT7
 EV - + -
PIK3CA
PIK3CA
KAT7
AKT
p-AKT (Ser473)
p-AKT (Ser473)
GAPDH
GAPDH
 BT549/IR
 BT549
 C
 D
Supplementary Figure. A-B, Effects of KAT7 knockdown on AKT phosphorylation and PIK3CA protein expression in breast cancer cells. C-D, PIK3CA overexpression rescued KAT7 knockdown-inhibited surviving fractions and cell proliferation after irradiation. EV represents empty vector. Error bars in C, and D represent the mean ± S.D. ***P < 0.001. Data are representative of three independent experiments.
